# Fundamental Motor Skill Delays in Preschool Children With Disabilities: 2012 National Youth Fitness Survey

**DOI:** 10.3389/fpubh.2021.758321

**Published:** 2021-12-07

**Authors:** E. Andrew Pitchford, Willie Leung, E. Kipling Webster

**Affiliations:** ^1^Department of Kinesiology, Iowa State University, Ames, IA, United States; ^2^Department of Health Sciences and Human Performance, University of Tampa, Tampa, FL, United States; ^3^Institute of Public and Preventive Health, Augusta University, Augusta, GA, United States

**Keywords:** motor development, pediatrics, gross motor, locomotor, object control, NHANES

## Abstract

Delays in fundamental motor skill (FMS) competency have been observed in a variety of children with disabilities. However, evidence of FMS delays is largely limited to small, geographically specific, limitedly diverse, and non-representative samples. The purpose of this study was to examine the association between FMS competency and reported disability status among pre-school children, ages 3–5 years, using the 2012 National Youth Fitness Survey (NYFS). In total, 329 preschool children (49% female; 4.00 ± 0.04 years of age) from the 2012 NYFS completed the Test of Gross Motor Development−2, including 43 preschoolers identified with a disability based on parental report (44% female; 4.20 ± 0.16 years). Associations were examined with logistic regression using sampling weights. Poor FMS competency, defined as gross motor quotient scores ≤ 79, was observed in significantly more children with disabilities (29%) than children without disabilities (10%, OR = 3.5, *p* = 0.04). While not statistically significant, there was a growing disparity in FMS competency at age 5 (41 vs. 11%) compared to age 3 (15 vs. 9%, OR = 1.80, *p* = 0.30). The results provide additional evidence for poor FMS competency among pre-school children with disabilities. FMS should be an early part of comprehensive assessments for all children suspected of disability or development delay as it is critical to identify and intervene upon FMS delays before discrepancies can widen.

## Introduction

Fundamental motor skills (FMS), including locomotor and object control skills, are the building blocks for developing advanced, complex motor skills to be used in heath-enhancing physical activities (1). FMS represent an important aspect of development during early childhood and a growing body of evidence suggests that motor competence plays a reciprocal role with physical activity to promote positive health in children (2, 3). This dynamic relationship between motor competence and physical activity is inversely related to obesity risk (2), and is positively interconnected with perceived motor competence and health-related physical fitness (2, 3). Evidence from multiple longitudinal studies shows higher motor competence in early childhood is significantly associated with higher levels of adolescent physical activity and physical fitness (4–6). Similar evidence has shown that children with higher FMS competency were more physically active as adults (7), highlighting the vital role FMS plays in promoting lifelong physical activity engagement. Conversely, delays in FMS competence during early childhood may be particularly problematic as poor FMS competency can lead to lower levels of physical activity across the life span (4, 6, 7), poorer health-related fitness (5, 8), decreased perceptions of competence (9), and higher risk of overweight (2, 10, 11). Not achieving a minimum level of proficiency in FMS may lead to children not attaining physical activity guidelines as they get older (12), which may further contribute to other negative health consequences including greater risk of heart disease, diabetes, and cancer (13). Multiple interventions, including programs targeting preschoolers, have shown efficacy in improving FMS competence (14), and provide evidence that gains in FMS competency are experience-dependent. Thus, it remains critical to identify and address low competence in FMS as early as possible.

Individuals with disabilities experience delays and deficits in FMS competency during early childhood, with significantly lower FMS competency reported in children with a range of disabilities (15–18). Concurrently, children with disabilities also engage in lower rates of physical activity (19–21), participate in fewer leisure and recreational activities (22–24), exhibit poor health-related physical fitness (25–27), and have higher proportions of children with overweight and obese weight status (28–30). Despite significant deficits and delays, multiple intervention studies have shown that children with disabilities can improve FMS competence if these skills are directly taught and practiced (31–33). Thus, identifying FMS delays in preschoolers with disabilities is particularly important so that early intervention to remediate these delays in FMS may begin.

The National Health and Nutrition Examination Survey (NHANES) National Youth Fitness Survey (NYFS) included the Test of Gross Motor Development−2 (TGMD-2) (34) as part of the 2012 examination for children 3–5 years of age (35). This is an important step in providing nationally representative data on FMS competency during the preschool years. Multiple analyses of these 2012 NYFS data have been published recently using TGMD-2 data (36–41). However, none of these analyses have addressed disability within the sample.

The purpose of this study was to examine the association between FMS competency and reported disability status among preschool children from the 2012 NYFS sample. The use of the NYFS permits these associations to be examined in a diverse, nationally representative sample; currently absent from the FMS literature. Differences between preschoolers with and without disability can provide context about the degree of FMS competency delays among preschoolers in the United States and help to identify additional factors associated with these delays. The study outcomes may also have implications for special education and early intervention services provided to qualifying children and families under the Individuals with Disabilities Education Act (IDEA) (42).

## Materials and Methods

This secondary data analysis utilized cross-sectional data from the 2012 NYFS, a multi-stage probability sample. As part of the 2012 NYFS, 1,640 youth, aged 3–15 years, were interviewed and 1,576 completed physical examinations (43). FMS competency was only examined in children aged 3–5 years (35). Thus, our analysis of FMS was limited to preschool-age children who completed the TGMD-2.

### Parental Reports

Parents completed survey questionnaires to report participant demographics including age (in years), sex, and race/ethnicity (43). Parents also responded to four questions regarding physical function. Specific questions included whether the child has an impairment or health problem (1) that limits ability walk, run, or play, (2) that has lasted, or is expected to last, 12 months or longer, (3) that requires use of special equipment, and (4) receives special education or early intervention services (44). For the purposes of this analysis, disability was defined as a positive response to at least one of the four questions. This approach is consistent with previous analyses of disability using NHANES databases (45, 46).

### FMS Competency

The TGMD-2 (34) is an assessment of FMS competency for children between the ages of 3 and 10 years. In short, the TGMD-2 includes 12 fundamental motor skills, including 6 locomotor skills (running, galloping, hopping, leaping, horizontal jumping, and sliding) and 6 object control skills (striking a stationary ball, stationary dribbling, catching, kicking, overhand throw, and underhand roll). Each skill has 3–5 criteria, predominately qualitative aspects of movement form, which are used to evaluate the proficiency of skill performance. Each performance criterion was evaluated via systematic, visual observation to determine if the criteria is present (1) or absent (0). Standardized scores, based on United States national normative data by age and sex (34), were calculated for the gross motor quotient (GMQ) and the locomotor and object control skill subscales. “Poor” performance can be interpreted from standardized subscale scores ≤ 5 and GMQ scores ≤ 79 (34). These scores are consistent with performance < 25th percentile, a common criteria used for identification of need in special education (47). For this analysis, children were classified dichotomously based on “poor” performance on each subscale and GMQ based on these standardized score cut-points (34). Additional detailed information on the TGMD-2 can be accessed through the test manual (34) and 2012 NYFS protocol (35).

All preschoolers followed a standardized protocol for TGMD-2 assessment including standard instructions and demonstrations from the assessor, a practice trial to check for understanding of the task, and two test trials. The test trials were scored live by trained and experienced assessors under the consultation of the TGMD-2 developer (Dale A. Ulrich, PhD) (35, 36). Of note, children with “physical limitations requiring a wheelchair; amputations of the leg, foot, arm, or hand; paralysis of one or both arms or hands; hand/arm/shoulder/leg surgery in last 3 months; or mental impairment” (35) were excluded from 2012 NYFS TGMD-2 data collection and would not be reflected in this sample. However, our analysis of these data did not identify any children with disabilities who were specifically excluded from completing the TGMD-2 based on these criteria. If children were excluded for this reason, they did not engage in any aspect of the 2012 NYFS.

### Statistical Analysis

Data for each test were accessed from the National Center for Health Statistics website and combined. Independent groups were created based on disability status. All statistical analyses were conducted using R (Vienna, Austria), with the “survey” package, accounting for the 2012 NYFS survey design with sample weights, primary sampling unit, and clustering variables. Descriptive statistics were calculated for demographics and FMS competency (i.e., standardized TGMD-2 scores) with both weighted and unweighted samples. Wald's chi-square was employed to examine associations in proportions of “poor” FMS between preschoolers with and without disabilities. Multiple logistic regression models were then tested to examine odds ratios (OR) of “poor” FMS between preschoolers with and without disabilities while accounting for covariates, including age (3/4/and 5 year olds), sex (male/female), and Hispanic ethnicity (yes/no). Alpha level was set at 0.05 for all analyses.

## Results

In total, 329 preschool children with complete data were selected for analysis (49% female; *M* = 4.00 ± 0.04 years of age), including 43 children with disabilities (44% female; *M* = 4.20 ± 0.16 years) and 286 children without (50% female, *M* = 4.00 ± 0.04 years). There were no statistically significant differences in demographics (i.e., sex, age, race/ethnicity) between groups (*p* > 0.05; see Table 1).

**Table 1:** Demographic characteristics of children with and without disabilities participating in the 2012 NYFS.

	**with Disabilities (*****n*** **= 43)**		**without Disabilities (*****n*** **= 286)**		**Total (*****n*** **= 329)**	
**Variables**	**Unweighted Sample Size**	**Proportion** **(95% CI)**	**Unweighted Sample Size**	**Proportion** **(95% CI)**	**Unweighted Sample Size**	**Proportion** **(95% CI)**
**Gross Motor Quotient, %**						
Poor (scores ≤ 79)	13	28.8 (12.0, 53.0)	30	10.0 (6.7, 15.0)	43	12.9 (91.5, 18.0)
Average (scores > 79)	30	71.8 (46.8, 88.0)	256	90.0 (85.2, 93.0)	286	87.2 (82.3, 91.0)
**Locomotor Skills, %**						
Poor (scores ≤ 5)	10	22.9 (9.5, 46.0)	18	6.4 (4.0, 10.0)	28	8.9 (6.54, 12.0)
Average (scores >5)	33	77.1 (54.3, 91.0)	268	93.6 (89.9, 96.0)	301	91.1 (87.9, 93.0)
**Object Control Skills, %**						
Poor (scores ≤ 5)	10	24.6 (10.8, 47.0)	20	7.1 (4.0, 12.0)	30	9.50 (6.91, 13.0)
Average (scores > 5)	33	75.4 (53.1, 89.0)	266	92.9 (87.8, 96.0)	299	90.5 (87.1, 93.0)
**Age, years** ^**a**^	43	4.2 ± 0.16	286	4.0 ± 0.06	329	4.0 ± 0.04
3 years old, %	10	21.6 (9.4, 42.0)	89	31.5 (24.7, 39.0)	99	30.0 (25.6, 35.0)
4 years old, %	18	41.1 (28.5, 55.0)	94	35.1 (28.3, 43.0)	112	36.1 (30.0, 43.0)
5 years old, %	15	37.3 (20.4, 58.0)	103	33.3 (2.6, 41.0)	118	33.9 (28.4, 40.0)
**Sex, %**						
Male	25	55.8 (39.4, 71.0)	145	49.6 (43.2, 56.0)	166	51.0 (46.2, 56.0)
Female	18	44.2 (29.0, 61.0)	145	50.4 (44.0, 57.0)	163	49.0 (44.3, 54.0)
**Hispanic, %**						
Hispanic	12	17.9 (8.9, 33.0)	98	25.6 (14.8, 40.0)	110	24.4 (14.0, 39.0)
Non-Hispanic	31	82.1 (67.5, 91.0)	188	74.4 (59.5, 85.0)	219	75.6 (61.0, 86.0)

*CI, confidence interval*.

a *Weighted Mean ± Standard Error*.

**Figure 1:**
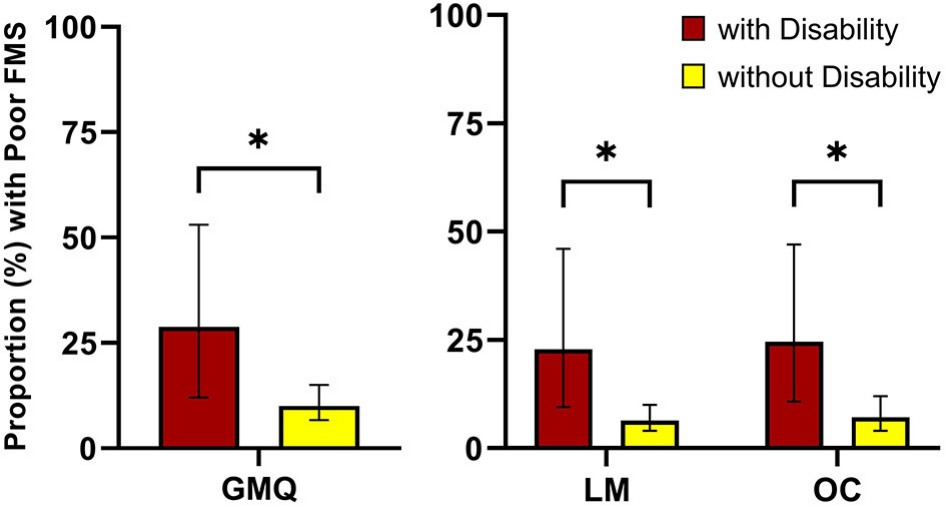
Gross motor quotient and subscale scores between children with and without disabilities. GMQ, gross motor quotient; LM, locomotor subscale; OC, object control subscale. *p ≤ 0.05.

Wald chi-square tests show that the proportion of preschoolers exhibiting “poor” FMS competency were significantly higher among preschoolers with disabilities compared to those without disabilities (see Figure 1, Table 2). Significant associations were observed for GMQ, locomotor, and object control skills (p <0.05). In total, 28.8% (95% CI: 12.0, 53.0) of children with disabilities compared to only 10.0% (95% CI: 6.7, 15.0) of children without disabilities (*p* = 0.03) exhibited “poor” GMQ totals.

**Table 2:** Chi-square analyses compare motor skills performance between children with and without disabilities.

**Motor Skill Performance**	**F**	**ndf**	**ddf**	***p-*value**
Gross Motor Quotient (≤ 79)	6.1	1	14	0.03^*^
Locomotor skills (≤ 5)	6.5	1	14	0.02^*^
Object Control skills (≤ 5)	6.2	1	14	0.03^*^

*All analyses were conducted with Rao & Scott adjustment. F, F-statistic; ndf, numerator degrees of freedom; ddf, denominator degrees of freedom*.

* *p ≤ 0.05, bolded*.

**Figure 2:**
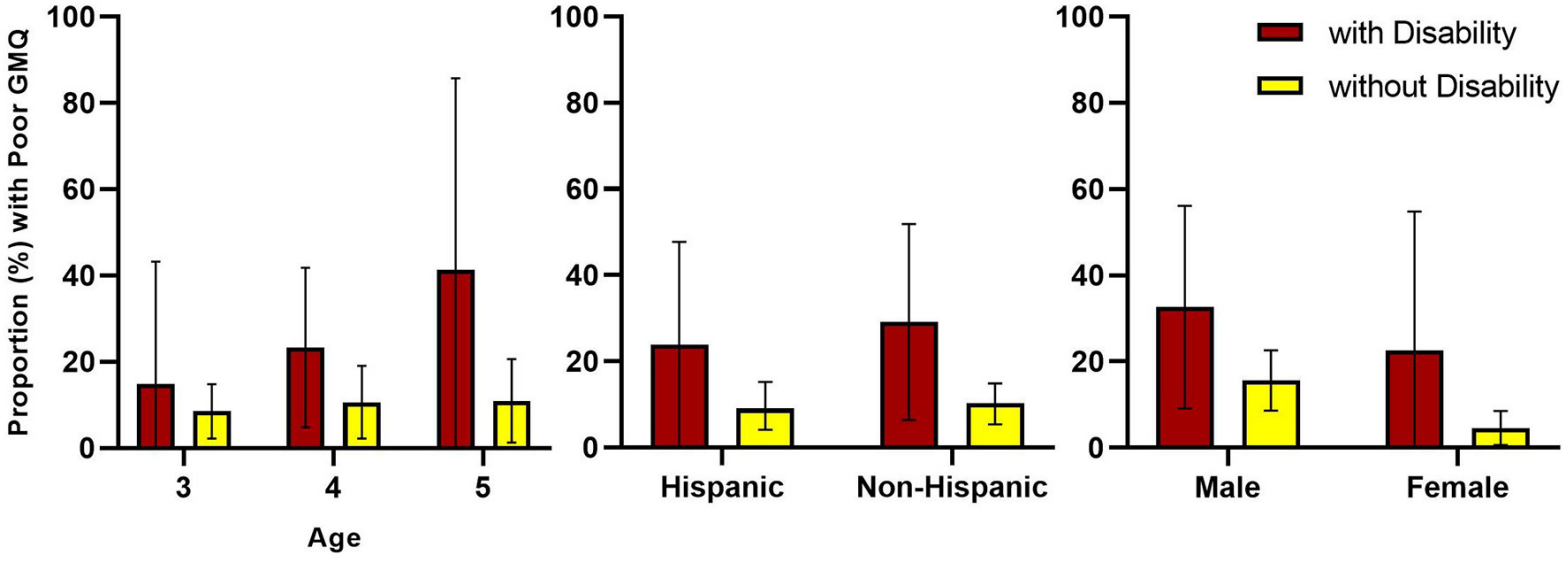
Proportion of poor motor competency between children with and without disabilities by age, Hispanic origin, and sex. GMQ, gross motor quotient.

Logistic regression models further analyzed associations between groups and demographic factors with FMS competency (see Table 3). Model 1 reflects the crude relationship between “poor” FMS and disability. The odds of children with disabilities exhibiting “poor” FMS competence based on GMQ were 3.5 times as high as the odds for children without disabilities (95% CI: 1.2, 10.0, *p* = 0.04). Models 2 through 4 reflect the addition of individual covariates (age, sex, and Hispanic origin, respectively). In each of these models, the effect of disability remained significant (*p* < 0.05) with consistent odds ratios. Notable associations with “poor” GMQ were identified for sex, but not age and Hispanic origin. The odds of “poor” GMQ in females was only 0.3 times the odds of males (95% CI: 0.09, 0.8, *p* = 0.03); however, significant associations were not identified for either age or Hispanic origin. Finally, model 5 reflects the fully adjusted model with all covariates. The odds of children with disabilities exhibiting “poor” GMQ increase by 3.4 times the odds of children without disabilities in having “poor” GMQ after adjusting for age, sex, and Hispanic origin, but the association was not statistically significant (95% CI: 1.1, 10.3, *p* = 0.06). Additionally, no covariate with significantly associated with “poor” GMQ, including female sex (OR: 0.3, 95% CI: 0.09, 0.8, *p* = 0.06). Descriptive trends, while not statistically significant, can be visually observed in the proportions of “poor” GMQ across these demographic factors (see Figure 2).

**Table 3:** Logistic regression of Gross Motor Quotient between children with and without disabilities.

**Poor Gross Motor Quotient (Standardized Scores ≤ 79)**															
**Variables**	**Model 1**			**Model 2**			**Model 3**			**Model 4**			**Model 5**		
	**OR**	**95% CI**	***p-*** **value**	**OR**	**95% CI**	***p-*** **value**	**OR**	**95% CI**	***p-*** **value**	**OR**	**95% CI**	***p-*** **value**	**OR**	**95% CI**	***p-*** **value**
**Disability**															
with Disabilities	**3.5**	**1.2, 10.0**	0.04^*^	**3.4**	**1.2, 9.6**	0.04^*^	**3.5**	**1.1, 10.4**	0.05^*^	**3.5**	**1.2, 10.1**	0.04^*^	3.4	1.1, 10.3	0.06
without Disabilities	1			1			1			1			1		
**Age**															
3 years old				1									1		
4 years old				1.3	0.4, 4.0	0.6							1.5	0.2, 2.7	0.5
5 years old				1.8	0.6, 4.8	0.3							1.9	0.3, 4.3	0.3
**Sex**															
Male							1						1		
Female							**0.3**	**0.1, 0.8**	0.03^*^				0.3	0.1, 0.8	0.06
**Ethnicity**															
Hispanic										0.9	0.4, 1.9	0.8	0.9	0.4, 2.0	0.6
Non-Hispanic										1			1		

*OR, odds ratio; CI, confidence interval*.

* *p ≤ 0.05, bolded. Model 1. Odd ratio from logistic regression model were computed form the outcome variable of poor gross motor quotient (GMQ) scores (≤ 79/>79) with the exposure variable of disabilities (with/without). Model 2. Odd ratio from logistic regression model were computed form the outcome variable of poor GMQ scores (≤ 79/>79) with the exposure variable of disabilities (with/without) adjusted for age (3 years old, 4 years old, 5 years old). Model 3. Odd ratio from logistic regression model were computed form the outcome variable of poor GMQ scores (≤ 79/>79) with the exposure variable of disabilities (with/without) adjusted for gender (male/female). Model 4. Odd ratio from logistic regression model were computed form the outcome variable of poor GMQ scores (≤ 79/>79) with the exposure variable of disabilities (with/without) adjusted for Hispanic status (yes/no). Model 5. Odd ratio from logistic regression model were computed form the outcome variable of poor GMQ scores (≤ 79/>79) with the exposure variable of disabilities (with/without) adjusted for age (3 years old, 4 years old, 5 years old), gender (male/female), and Hispanic status (yes/no)*.

Similarly high odds ratios were observed for the locomotor (OR: 4.3, 95% CI: 1.3, 14.5, *p* = 0.03) and object control (OR: 4.3, 95% CI: 1.3, 14.6, *p* = 0.04) subscales. Full logistic regression models for locomotor and object control subscales are provided in Supplementary Tables 1, 2 and full proportions and 95% confidence intervals across demographic factors are provided in Supplementary Table 3.

## Discussion

The purpose of this secondary data analysis was to compare FMS competency between preschoolers in the United States, both with and without disabilities, based on the 2012 NYFS. Statistically significant associations with moderate to large odds ratios in “poor” TGMD-2 GMQ scores were observed between groups. Preschoolers with disabilities consistently had higher proportions of “poor” FMS competency compared to preschoolers without disabilities. These findings from a nationally representative sample provide additional evidence that children with identified disabilities are likely to exhibit delays in motor development before entering formal education (e.g., kindergarten, elementary school). These delays in FMS competency are consistent with eligibility for adapted physical education services under IDEA (42, 47, 48).

Substantial associative relationships were observed between disability groups for the total GMQ, as well as the locomotor and object control subscales. Children with disabilities were more than 3.5 times more likely to have “poor” FMS competency, while accounting for age, sex, and ethnicity. More importantly, 28% of children with disabilities had “poor” GMQ scores consistent with a developmental motor delay (i.e., < 25th percentile) (42). The 12.9% of preschoolers in the total sample, including the 10% of children without disabilities, which demonstrated “poor” FMS competence, is close, but slightly higher than the rate expected from the TGMD-2 normative sample (9.2%) (34). This could be due to discrepancies between the population in 2000 reflected in the normative sample (34) and the population in the 2012 NYFS (43). The Test of Gross Motor Development−3 (49) has since been published with updated normative standards. Recent data comparing concurrent TGMD-3 and TGMD-2 scores have identified discrepancies (50, 51), with higher normative scores on the TGMD-3 compared to the TGMD-2. This could suggest that population-level FMS competency has decreased over the last decade, but additional studies are needed to properly address that issue. Regardless, this rate suggests there is a sizable population of preschoolers reflected in the nationally representative 2012 NYFS sample, with delays in FMS development that could benefit from remediation or early intervention services. While enrollment in special education or early intervention services was one of the four criteria used to define disability for this analysis, it is unknown what services were received and how long children may have been enrolled or receiving these services.

Additional demographic factors were also examined to better understand associations in FMS competency between preschoolers with and without disability. The only demographic factor that reached statistical significance in any model was sex, with females having 0.30 times the odds of males to exhibit “poor” GMQ scores. Males, both with and without disabilities, are typically found to be more advanced than females in FMS competency, particularly in object control skills (16, 52, 53). This relationship is likely due to boys receiving more encouragement toward object control and ball skills compared to girls, while biological factors are unlikely to be a factor in prepubescent children (52, 53). Within the 2012 NYFS sample, males had significantly higher object control raw scores compared to females (36). However, a unique result in our analysis was females having lower odds of “poor” GMQ. This discrepancy is likely due to the relative comparison (e.g., norm-referenced) of FMS competency in the current analysis, compared to absolute comparisons. For example, the TGMD-2 normative standards defined “poor” object control skills for a 5-year-old male as a score ≤ 17, but ≤ 14 for a 5-year old female (34). Coupled with significantly greater locomotor raw scores among girls compared to boys (36), a subscale with one set of normative standards for male and females (34), could explain the lower rates of “poor” FMS competency of females, regardless of disability.

In addition to the statistically significant sex effect, additional trends of interest were observed across age and Hispanic origin. The proportions shown in Figure 2 consistently reflect the higher risk of “poor” FMS competency in children with disabilities, but also show a growing disparity with increasing age. While not statistically significant, 5-year-old children had 1.8 times greater odds of “poor” GMQ scores compared to 3-year-old children. While multiple studies of children with developmental disabilities have shown substantial delays in FMS competency (54, 55), the potential for a widening delay with age among preschoolers is concerning. These results indicate that waiting until traditional public schooling (i.e., kindergarten) to assess for FMS competency may allow disparities to increase. Hispanic origin was also not a significant factor in any of the models, but it appears that “poor” FMS competency was higher in non-Hispanic children, especially those with disabilities. Zhang et al. (41) also reported slightly higher raw TGMD-2 scores in Hispanic children compared to non-Hispanic children from the 2012 NYFS sample.

There is limited research on the pervasiveness of developmental motor delays among preschoolers in the United States. However, our results from the 2012 NYFS are similar, but not identical, to a recent multi-site study that also utilized the TGMD-2 to measure FMS competency in preschoolers (56). FMS competency was examined in 580 children 3–6 years of age, compiled from seven early learning centers in five states. Using a similar definition of GMQ < 25th percentile as an indicator for developmental motor delays, Brian et al. (56) reported that 77% of the sample had below average FMS competency. The proportion of “poor” FMS competency observed in the current study, 28%, is large, but also meaningfully lower than Brian et al.'s report (56). Differences could be due to the samples, although both studies include multi-state samples with diverse demographics; however, the Brian et al. (56) sample included approximately 71% of children from low socioeconomic backgrounds. A separate analysis of the 2012 NYFS data identified 39% of children to be below the poverty line (39). Previous research has shown that children from a disadvantaged environment are likely to be at-risk for developmental delay in FMS, but are also receptive to improvements in these skills through early intervention programs targeting FMS (57, 58).

It is critical to intervene early to address delays in FMS competency. A wealth of intervention research is available to improve FMS in preschool children, both with and without disabilities. In the general population, most published intervention studies, including interventions targeting preschoolers, have reported significant increases in FMS competency (14). Similar findings have also been reported for interventions targeting preschoolers with developmental delays (59) and developmental disabilities (31, 32, 60). Pertinent intervention variables for children with disabilities appear to be the duration (i.e., longer than 16 weeks vs. shorter programs) and location of the intervention (i.e., controlled settings vs. school/practical settings) (31). However, most research on young children with disabilities, including interventions, is limited by small sample sizes, inconsistent measures, simple research designs, and limited use of theoretical foundations (31, 32, 59, 60). The pervasiveness of early FMS delays demonstrated in the 2012 NYFS data suggest that more effort should be put into generating appropriate, targeted, adequately powered, and translatable intervention studies for this population.

Multiple limitations in the present 2012 NYFS data and secondary analysis should be acknowledged and considered when interpreting results. The primary limitation of these data is that specific disability diagnoses or descriptors were not available. Disability was defined by parental report of four questions and reflects a proxy of disability status. Further, data on the type of services (e.g., physical and occupational therapy) received were not provided. Given the exclusion criteria for completing the TGMD-2 in the 2012 NYFS, the children included in this sample likely reflect general developmental delays, as opposed to significant intellectual or physical disabilities. Despite the limitation, this sample is consistent with the demographics of preschoolers receiving early services in 2012 (61).

An additional limitation is the sample size. While the study benefits from the use of a nationally representative sample, the final sample of 329 preschoolers including 43 children identified with a disability, remains relatively small. Other reports of these data had a similar sample size (36–41), but the large discrepancy in sample size between comparison groups could skew results. However, the study is strengthened by use of diverse, national representative sample from NHANES and identifies useful information about the comorbidity of motor delays in preschoolers with disabilities using survey design approach.

Finally, it is notable that the data used for this analysis are derived from the 2012 NYFS, which is nearing a decade old. Thus, it could be argued that the results may not be reflective of children today. Although this data may be dated, it represents the only national survey of FMS competency in preschoolers. These TGMD-2 data have been used in a small body of literature (36–41), but are relatively underutilized compared to other NYFS components (e.g., physical fitness, weight status). We believe it is important that TGMD-2 data from the 2012 NYFS be utilized to the greatest extent possible to encourage NHANES to evaluate FMS in future surveys.

## Conclusion

In conclusion, significant delays in FMS competency were identified among preschool children with disabilities compared to preschoolers without disabilities. The identification of low FMS competency among preschool children provides evidence that adapted physical education services are warranted for early intervention. Federal law mandates that special education services be provided to children with disabilities from birth to age 21 (42). A significant delay in FMS competency for a child with or at-risk for developmental delays is sufficient to receive adapted physical education as part of the child's individualized special education program (47, 48). The disparities documented in this national sample suggest that a large proportion of preschool children with disabilities should be eligible for services.

## Data Availability Statement

Publicly available datasets were analyzed in this study. This data can be found here: https://wwwn.cdc.gov/nchs/nhanes/search/nnyfs12.aspx.

## Ethics Statement

The studies involving human participants were reviewed and approved by National Center for Health Statistics Ethics Review Board. Written informed consent to participate in this study was provided by the participant's legal guardian/next of kin.

## Author Contributions

EP and EW: conceptualization and methodology. WL: formal analysis. EP and WL: data curation. EP: visualization and writing. EP, WL, and EW: review and editing. All authors have read and agreed to the published version of the manuscript.

## Conflict of Interest

The authors declare that the research was conducted in the absence of any commercial or financial relationships that could be construed as a potential conflict of interest.

## Publisher's Note

All claims expressed in this article are solely those of the authors and do not necessarily represent those of their affiliated organizations, or those of the publisher, the editors and the reviewers. Any product that may be evaluated in this article, or claim that may be made by its manufacturer, is not guaranteed or endorsed by the publisher.
