# Supplementary material for: Fundamental Motor Skill Delays in Preschool Children With Disabilities: 2012 National Youth Fitness Survey
Source: Front Public Health. 2021 Dec 7;9:758321. doi: 10.3389/fpubh.2021.758321 (PMC8696669; doi:10.3389/fpubh.2021.758321)
Supplement: Supplementary file 1 [file Data_Sheet_1.docx]

**Table S1.** Logistic regression of locomotor skills between children with and without disabilities

| **Poor Locomotor Skills (Standardized Scores ≤5)** | | | | | | | | | | | | | | | |
| --- | --- | --- | --- | --- | --- | --- | --- | --- | --- | --- | --- | --- | --- | --- | --- |
| **Variables** | **Model 1** | | | **Model 2** | | | **Model 3** | | | **Model 4** | | | **Model 5** | | |
|  | OR | 95% CI | *p* | OR | 95% CI | *p* | OR | 95% CI | *p* | OR | 95% CI | *p* | OR | 95% CI | *p* |
| **Disability** |  |  |  |  |  |  |  |  |  |  |  |  |  |  |  |
| with Disabilities | **4.3** | **1.3, 14.5** | **0.03*** | **4.5** | **1.4, 14.4** | **0.03*** | **4.3** | **1.2, 15.5** | **0.05*** | **4.3** | **1.3, 14.2** | **0.03*** | **4.3** | **1.2, 15.7** | **0.05*** |
| without Disabilities | 1 |  |  | 1 |  |  | 1 |  |  | 1 |  |  | 1 |  |  |
| **Age** |  |  |  |  |  |  |  |  |  |  |  |  |  |  |  |
| 3 years old |  |  |  | 1 |  |  |  |  |  |  |  |  | 1 |  |  |
| 4 years old |  |  |  | 0.7 | 0.2, 2.4 | 0.5 |  |  |  |  |  |  | 0.7 | 0.2, 2.7 | 0.7 |
| 5 years old |  |  |  | 1.0 | 0.3, 3.4 | 1.0 |  |  |  |  |  |  | 1.1 | 0.3, 4.3 | 0.9 |
| **Sex** |  |  |  |  |  |  |  |  |  |  |  |  |  |  |  |
| Male |  |  |  |  |  |  | 1 |  |  |  |  |  | 1 |  |  |
| Female |  |  |  |  |  |  | **0.3** | **0.1, 0.8** | **0.03*** |  |  |  | **0.3** | **0.1, 0.8** | **0.04*** |
| **Ethnicity** |  |  |  |  |  |  |  |  |  |  |  |  |  |  |  |
| Hispanic |  |  |  |  |  |  |  |  |  | 0.9 | 0.4, 1.9 | 0.8 | 0.9 | 0.4, 2.0 | 0.8 |
| Non-Hispanic |  |  |  |  |  |  |  |  |  | 1 |  |  | 1 |  |  |

*Abbreviations.* OR, odds ratio; CI, confidence interval; *p*, *p*-value.

**p* ≤ 0.05, **bolded**.

Model 1. Odd ratio from logistic regression model were computed form the outcome variable of poor locomotor (LM) scores (≤5/>5) with the exposure variable of disabilities (with/without).

Model 2. Odd ratio from logistic regression model were computed form the outcome variable of poor LM scores (≤5/>5) with the exposure variable of disabilities (with/without) adjusted for age (3 years old, 4 years old, 5 years old).

Model 3. Odd ratio from logistic regression model were computed form the outcome variable of poor LM scores (≤5/>5) with the exposure variable of disabilities (with/without) adjusted for gender (male/female).

Model 4. Odd ratio from logistic regression model were computed form the outcome variable of poor LM scores (≤5/>5) with the exposure variable of disabilities (with/without) adjusted for Hispanic status (yes/no).

Model 5. Odd ratio from logistic regression model were computed form the outcome variable of poor LM scores (≤5/>5) with the exposure variable of disabilities (with/without) adjusted for age (3 years old, 4 years old, 5 years old), gender (male/female), and Hispanic status (yes/no).

**Table S2.** Logistic regression of object control skills between children with and without disabilities

| **Poor Object Control Skills ( Standardized Scores ≤5)** | | | | | | | | | | | | | | | | | | |
| --- | --- | --- | --- | --- | --- | --- | --- | --- | --- | --- | --- | --- | --- | --- | --- | --- | --- | --- |
| **Variables** | **Model 1** | | | **Model 2** | | | **Model 3** | | | | **Model 4** | | | | **Model 5** | | | |
|  | OR | 95% CI | *p* | OR | 95% CI | *p* | OR | 95% CI | *p* | OR | | 95% CI | *p* | OR | | 95% CI | *p* |  |
| **Disability** |  |  |  |  |  |  |  |  |  |  | |  |  |  | |  |  |  |
| with Disabilities | **4.3** | **1.3, 14.6** | **0.04*** | **4.1** | **1.2, 14.5** | **0.05*** | **4.2** | **1.3, 13.9** | **0.04*** | **4.2** | | **1.2, 14.1** | **0.04*** | 3.9 | | 1.1, 13.8 | 0.06 |  |
| without Disabilities | 1 |  |  | 1 |  |  | 1 |  |  | 1 | |  |  | 1 | |  |  |  |
| **Age** |  |  |  |  |  |  |  |  |  |  | |  |  |  | |  |  |  |
| 3 years old |  |  |  | 1 |  |  |  |  |  |  | |  |  | 1 | |  |  |  |
| 4 years old |  |  |  | 1.5 | 0.4, 5.1 | 0.5 |  |  |  |  | |  |  | 1.6 | | 0.5, 5.1 | 0.4 |  |
| 5 years old |  |  |  | 1.9 | 0.6, 6.0 | 0.3 |  |  |  |  | |  |  | 2.1 | | 0.6, 7.0 | 0.3 |  |
| **Sex** |  |  |  |  |  |  |  |  |  |  | |  |  |  | |  |  |  |
| Male |  |  |  |  |  |  | 1 |  |  |  | |  |  | 1 | |  |  |  |
| Female |  |  |  |  |  |  | 0.4 | 0.2, 1.0 | 0.08 |  | |  |  | 0.4 | | 0.2, 0.9 | 0.06 |  |
| **Ethnicity** |  |  |  |  |  |  |  |  |  |  | |  |  |  | |  |  |  |
| Hispanic |  |  |  |  |  |  |  |  |  | 0.6 | | 0.3, 1.5 | 0.3 | 0.6 | | 0.2, 1.5 | 0.3 |  |
| Non-Hispanic |  |  |  |  |  |  |  |  |  | 1 | |  |  | 1 | |  |  |  |

*Abbreviations.* OR, odds ratio; CI, confidence interval; *p*, *p*-value.

**p* ≤ 0.05, **bolded**.

Model 1. Odd ratio from logistic regression model were computed form the outcome variable of poor object control (OC) scores (≤5/>5) with the exposure variable of disabilities (with/without).

Model 2. Odd ratio from logistic regression model were computed form the outcome variable of poor OC scores (≤5/>5) with the exposure variable of disabilities (with/without) adjusted for age (3 years old, 4 years old, 5 years old).

Model 3. Odd ratio from logistic regression model were computed form the outcome variable of poor OC scores (≤5/>5) with the exposure variable of disabilities (with/without) adjusted for gender (male/female).

Model 4. Odd ratio from logistic regression model were computed form the outcome variable of poor OC scores (≤5/>5) with the exposure variable of disabilities (with/without) adjusted for Hispanic status (yes/no).

Model 5. Odd ratio from logistic regression model were computed form the outcome variable of poor OC scores (≤5/>5) with the exposure variable of disabilities (with/without) adjusted for age (3 years old, 4 years old, 5 years old), gender (male/female), and Hispanic status (yes/no).

**Table S3.** Proportions of poor and average GMQ of preschoolers with and without disabilities by demographic factors

|  | **with Disability** | | | | **without Disability** | | | |
| --- | --- | --- | --- | --- | --- | --- | --- | --- |
|  | **Poor GMQ (≤79)** | | **Average GMQ (>79)** | | **Poor GMQ (≤79)** | | **Average GMQ (>79)** | |
|  | Unweighted  *n* | Proportion  (95% CI) | Unweighted *n* | Proportion  (95% CI) | Unweighted *n* | Proportion  (95% CI) | Unweighted *n* | Proportion  (95% CI) |
| **Age, %** |  |  |  |  |  |  |  |  |
| 3 years old | 2 | 14.8 (-13.6, 43.2) | 8 | 85.2 (56.8, 113.6) | 6 | 8.5 (2.2, 14.8) | 83 | 91.5 (85.2, 97.8) |
| 4 years old | 4 | 23.3 (4.8, 41.8) | 14 | 76.7 (58.2, 95.2) | 13 | 10.6 (2.2, 19.0) | 81 | 89.4 (81.0, 97.8) |
| 5 years old | 7 | 41.3 (-3.0, 85.7) | 8 | 58.7 (14.3, 103.0) | 11 | 10.9 (1.3, 20.6) | 92 | 89.1 (79.4, 98.7) |
| **Sex, %** |  |  |  |  |  |  |  |  |
| Male | 8 | 32.6 (9.1, 56.1) | 17 | 67.4 (43.9, 90.9) | 22 | 15.6 (8.6, 22.6) | 119 | 84.4 (77.4, 91.4) |
| Female | 5 | 22.6 (-9.5, 54.8) | 13 | 77.4 (45.2, 109.5) | 8 | 4.6 (0.6, 8.5) | 137 | 95.4 (91.5, 99.4) |
| **Hispanic, %** |  |  |  |  |  |  |  |  |
| Hispanic | 3 | 23.8 (-0.1, 47.7) | 9 | 76.2 (52.3, 100.0) | 11 | 9.7 (4.1, 15.2) | 87 | 90.3 (84.8, 95.9) |
| Non-Hispanic | 10 | 29.2 (6.4, 51.9) | 21 | 70.8 (48.1, 93.6) | 19 | 10.2 (5.4, 14.9) | 169 | 89.8 (85.1, 94.6) |

*Abbreviations.* CI, confidence interval.
